# Treatment patterns, effectiveness, and patient‐reported outcomes of palbociclib therapy in Chinese patients with advanced breast cancer: A multicenter ambispective real‐world study

**DOI:** 10.1002/cam4.4767

**Published:** 2022-04-25

**Authors:** Lesang Shen, Jun Zhou, Yiding Chen, Jinhua Ding, Haiyan Wei, Jian Liu, Wenjie Xia, Bojian Xie, Xiaohong Xie, Xujun Li, Yuechu Dai, Guobing Zhang, Xia Qiu, Chao Li, Shanshan Sun, Wuzhen Chen, Dihe Gong, Hengyu Li, Jian Huang, Xia Jiang, Chao Ni

**Affiliations:** ^1^ Department of Breast Surgery Second Affiliated Hospital of Zhejiang University Hangzhou China; ^2^ Key Laboratory of Tumor Microenvironment and Immune Therapy of Zhejiang Province Hangzhou China; ^3^ Department of Breast Surgery Affiliated Hangzhou First People's Hospital of Zhejiang University Hangzhou China; ^4^ Department of Breast Surgery Ningbo Medical Center Lihuili Hospital Ningbo China; ^5^ Department of Breast Surgery First Affiliated Hospital of Zhejiang University Hangzhou China; ^6^ Department of Breast Surgery Zhejiang Provincial People's Hospital Hangzhou China; ^7^ Department of Breast Surgery Taizhou Enze Medical Center Enze Hospital Taizhou China; ^8^ Department of Breast Surgery First Affiliated Hospital of Zhejiang Chinese Medical University Hangzhou China; ^9^ Department of Breast Surgery Hwa Mei Hospital University of Chinese Academy of Sciences Ningbo China; ^10^ Department of Breast Surgery Taizhou Municipal Hospital Taizhou; ^11^ Quality Management Office Zhejiang Provincial People's Hospital Hangzhou China; ^12^ Department of Breast Surgery Zhoushan Hospital of Zhejiang University Zhoushan China; ^13^ Department of Breast Surgery Ningbo Hangzhou Bay Hospital Ningbo China; ^14^ Department of General Surgery Affiliated Changhai Hospital of The Second Military Medical University Shanghai China; ^15^ Department of Nutrition West China School of Public Health and West China Fourth Hospital, Sichuan University Chengdu China; ^16^ Department of Clinical Neuroscience, Centre for Molecular Medicine Karolinska Institute Stockholm Sweden

**Keywords:** adverse events, endocrine therapy, metastatic breast cancer, palbociclib, patient‐reported outcomes, real‐world study

## Abstract

**Background:**

Palbociclib was the only available cyclin‐dependent kinase 4/6 inhibitor in China until very recently, and its effect has not been systemically evaluated among Chinese patients. This study aims to assess the efficacy, safety and patient‐reported outcomes (PROs) of palbociclib plus endocrine therapy (ET) in real‐world China.

**Methods:**

An ambispective cohort study was conducted on patients with advanced HR+HER2− breast cancer who received palbociclib between July 2018, and November 2020 and were enrolled from 12 hospitals. Treatment patterns, survival outcomes, and safety events were documented, and PROs (European Organization for Research and Treatment of Cancer Quality of Life Questionnaire‐Core 30 items [EORTC QLQ‐C30] and EuroQoL 5 dimensions [EQ‐5D]) were analyzed. The Kaplan–Meier method was used to visualize and estimate the median progression‐free survival (mPFS). Log‐rank tests, Cox regressions, *t* tests, and chi‐square tests were performed for comparison.

**Results:**

A total of 190 patients (median follow‐up of 18.0 months) were enrolled. Palbociclib was mostly combined with aromatase inhibitors (66.3%), fulvestrant (32.6%), and tamoxifen (1.1%). The mPFS values were 21.0, 14.0, and 7.0 months with palbociclib administered in first‐ (*n* = 83), second‐ (*n* = 41) and subsequent‐line settings (*n* = 66), respectively. Endocrine sensitivity was significantly associated with patient prognosis (mPFS: 23.0, 12.0, and 6.0 months for endocrine naïve, acquired, and primary resistant patients, respectively, *p* < 0.01). The outcome was worse for patients who failed to meet the inclusion criteria of PALOMA‐3 than for those who met the criteria (later‐line: 6.0 months vs. 9.0 months). The most common adverse events (AEs) were neutropenia (74.2%; grade 3/4: 30.0%), fatigue (48.4%), anemia (32.6%), and thrombocytopenia (22.1%). PRO data suggested that palbociclib plus ET significantly improved cognitive and emotional function, pain symptoms, and overall quality of life.

**Conclusions:**

Palbociclib is effective for front‐line use and for treating endocrine‐sensitive patients in real‐world China and is generally well tolerated. The prevalence of AEs in the Chinese population is different from that reported in the PALOMA‐2/3 trials.

## Graphical abstract

Firstly, the adverse events of palbociclib among Chinese patients are different from those reported in clinical trials, as a lower rate of grade 3/4 neutropenia and a higher rate of anemia and thrombocytopenia are observed. Secondly, palbociclib is more effective for front lines and endocrine sensitive patients, but less effective for patients who are primary endocrine resistant and have received chemotherapy for metastatic disease. Thirdly, palbociclib plus aromatase inhibitor and palbociclib plus fulvestrant show similar efficacy irrespective of the treatment lines or previously received aromatase inhibitor.

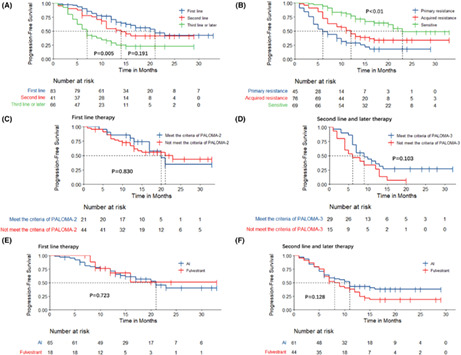

## 1 INTRODUCTION

Breast cancer (BC) is the most commonly diagnosed cancer worldwide, and nearly 1/5 of the total cases occur in China.^1^ Almost 70% of BCs are hormone receptor‐positive (HR+), a subtype in which endocrine therapy (ET) is the core treatment even among patients with metastatic disease.^2^ However, no substantial improvement in survival was achieved before the advent of cyclin‐dependent kinase 4/6 inhibitor (CDK4/6i) therapy.^3^, ^4^, ^5^ According to the results of the phase II PALOMA‐1 trial, palbociclib (a CDK4/6i) combined with letrozole as first‐line treatment for HR+ human epidermal growth factor receptor 2‐negative (HER2−) metastatic BC (MBC) patients almost doubled the median progression‐free survival (mPFS).^6^ Subsequent phase III trials have also confirmed that CDK4/6i greatly improves the prognosis of both ET‐naïve and ET‐resistant patients.^7^, ^8^, ^9^, ^10^, ^11^, ^12^, ^13^

Palbociclib (IBRANCE®) was approved by the China FDA in July 2018 and became the only CDK4/6i available in China until April 2021.^14^ However, as Asian participants were underrepresented in previous trials (14.6% in PALOMA‐2 and 21.3% in PALOMA‐3),^7^, ^8^ the efficacy and safety of palbociclib have not been systemically evaluated among Chinese patients. In addition, high cost (~26,000 USD/year) also hinders the standard administration of palbociclib in real‐world China.

Therefore, to comprehensively assess real‐world MBC patients in China treated with palbociclib plus ET, including their basic characteristics, safety events, prognosis, patient‐reported outcomes (PROs), and physician practice, we conducted a multicenter ambispective‐cohort‐based real‐world study (RWS) involving 190 Chinese MBC patients from 12 hospitals. We anticipate that our results will provide deep insights into the efficacy of palbociclib in Asian MBC patients and eventually aid in clinical decision‐making.

## 2 METHODS

### 2.1 Patients

An ambispective multicenter cohort study was performed involving patients with advanced BC (ABC) or MBC who received palbociclib from July 2018 to November 2020 and recruited from 12 participating hospitals in southeast China (full list in Data S1). Patients were followed until the end of October 2021. Ethical approval was obtained from the ethics committee of each participating hospital before the initiation of the study.

Inclusion criteria were defined as follows: diagnosis of ABC or MBC; confirmed HR+ and HER2− status; previous palbociclib treatment for at least 1 month in combination with any ET, regardless of lines of treatment; and regular follow‐up. Patient clinical data, including demographic, clinicopathologic, and treatment‐related information, were extracted from electronic medical records. Toxicity data and instances of dose reduction and interruption and discontinuation of therapy were recorded in the clinician documentation during scheduled visits and/or telephone encounters.

### 2.2 Outcomes

The primary outcome was PFS, defined as the time from the start of palbociclib until disease progression assessed by the treating physician according to RECIST version 1.1,^15^ death during the study, or end of follow‐up, whichever came first. Secondary outcomes included assessment of adverse events (AEs), recognized based on the Common Terminology Criteria for Adverse Events (CTCAE version 5.0), as well as overall survival (OS), defined as the time from treatment initiation to death or end of follow‐up. Endocrine sensitivity was defined based on time to recurrence. Acquired resistance to ET was defined as recurrence after 2 years of adjuvant therapy, recurrence within the first year after completion, or progression over 6 months after the last palliative ET treatment. Primary resistance was defined as relapse within 2 years of adjuvant therapy or progression within 6 months after the last palliative ET treatment. Patients who were diagnosed with de novo metastatic disease or recurrence 1 year after the completion of adjuvant therapy were considered ET sensitive.^2^

Patient‐reported outcomes were assessed using the European Organization for Research and Treatment of Cancer Quality of Life Questionnaire‐Core 30 items (EORTC QLQ‐C30)^16^ as well as the EuroQoL 5 dimensions (EQ‐5D) questionnaire^17^ at both baseline (retrospectively) and the end of March 2021 (Data S1). The questionnaires were gathered by scheduled clinic visits, telephone encounters, or the Electronic Questionnaire Star tool (http://www.wjx.cn/) via WeChat, a popular instant message app in China, and the process of completing the questionnaires of all patients was performed under the guidance of physicians.

### 2.3 Statistical analysis

Patient characteristics are presented as medians and ranges for continuous variables and as frequencies and percentages for categorical variables. Survival data were visualized using Kaplan–Meier curves and compared by the log‐rank test. Hazard ratios (HRs) and 95% confidence intervals (CIs) were also calculated using Cox proportional hazards models. Comparison of PROs was performed by using pairwise *t* tests (continuous variables) or chi‐square tests (categorical variables). Statistical significance was defined as two‐sided *p* < 0.05. All statistical analyses were performed using R software version 4.0.3.

We further summarized all currently available RWSs conducted for palbociclib plus ET in MBC through a meta‐analysis (the final search was conducted on April 20, 2021). The search strategy, inclusion, and exclusion criteria of the literature as well as analytical approaches are shown in Data S1.

## 3 RESULTS

### 3.1 Patient characteristics

A total of 190 patients treated with palbociclib plus ET were identified and enrolled in our study [median age at treatment initiation: 56 (range, 23–88) years]. Most patients were postmenopausal (67.4%) and had an Eastern Cooperative Oncology Group (ECOG) performance status of 0–1 (90.0%). Baseline characteristics are listed in Table 1. As a typical RWS, our study focuses on confirmed estrogen receptor (ER)+HER2− BC with locally recurrent or metastatic disease; specifically, 87.4% of our patients were ER+ progesterone receptor (PR)+, while 12.6% were ER+PR–. When palbociclib was started, 37 patients (19.5%) were diagnosed with de novo metastatic disease, 117 patients (61.6%) had visceral metastasis, 35 patients (18.4%) had bone‐only metastasis, and nine patients (4.7%) had brain metastasis. A total of 40.5% of our patients had a single metastatic site, while 27.9% had multiple (≥3) metastatic sites.

**TABLE 1:** Baseline patient characteristics of the real‐world Chinese population used and the PALOMA‐2/3 study population

	Total (*N =* 190)	First line (*N =* 83)	≥Second line (*N =* 107)	PALOMA‐2 (*N =* 444)^a^	PALOMA‐3 (*N =* 347)^a^
Median age (range), years	56 (23–88)	54 (23–88)	57 (29–86)	62 (30–89)	57 (30–88)
Median BMI (range)	22.96 (15.21–32.45)	22.76 (15.21–27.77)	23.23 (16.23–32.45)	NA	NA
ECOG performance status *N* (%)					
0–1	171 (90.0)	79 (95.2)	92 (86.0)	435 (98.0)	347 (100.0)
2–3	19 (10.0)	4 (2.1)	15 (14.0)	9 (2.0)	0 (0.0)
Menopausal status *N* (%)					
Postmenopausal	128 (67.4)	50 (60.2)	78 (72.9)	444 (100.0)	275 (79.3)
Premenopausal	62 (32.6)	33 (39.8)	29 (27.1)	0 (0.0)	72 (20.7)
Estrogen receptor *N* (%)					
Positive	190 (100.0)	83 (100.0)	107 (100.0)	444 (100.0)	347 (100.0)
Negative	0 (0.0)	0 (0.0)	0 (0.0)	0 (0.0)	0 (0.0)
Progesterone receptor *N* (%)					
Positive	166 (87.4)	75 (90.4)	91 (85.0)	NA	256 (73.8)
Negative	24 (12.6)	8 (9.6)	16 (15.0)	NA	91 (26.2)
Stage at initial diagnosis *N* (%)					
I	20 (10.5)	9 (4.7)	11 (10.3)	51 (11.5)	25 (7.2)
II	81 (42.6)	32 (38.6)	49 (45.8)	137 (30.9)	119 (34.3)
III	52 (27.4)	19 (22.9)	33 (30.8)	72 (16.2)	69 (19.9)
IV	37 (19.5)	23 (27.7)	14 (13.1)	138 (31.1)	67 (19.3)
Prior neoadjuvant and/or adjuvant chemotherapy *N* (%)					
Yes	125 (65.8)	47 (56.6)	78 (72.9)	213 (48.0)	NA
No	65 (34.2)	36 (43.4)	29 (27.1)	231 (52.0)	NA
Adjuvant endocrine therapy *N* (%)					
Tamoxifen	100 (52.6)	40 (48.2)	60 (56.1)	209 (47.1)	NA
AI	57 (30.0)	22 (26.5)	35 (32.7)	122 (27.5)	NA
None	33 (17.4)	21 (25.3)	12 (11.2)	113 (25.5)	NA
Endocrine sensitivity *N* (%)					
Sensitive^b^	69 (36.3)	46 (55.4)	23 (21.5)	345 (100.0)	0 (0.0)
Acquired resistance	76 (40.0)	24 (28.9)	52 (48.6)	0 (0.0)	274 (79.0)
Primary resistance	45 (23.7)	12 (14.5)	33 (30.8)	0 (0.0)	73 (21.0)
Number of metastatic sites *N* (%)					
1	77 (40.5)	49 (59.0)	28 (26.2)	138 (31.1)	111 (32.0)
2	60 (31.6)	19 (22.9)	41 (38.3)	NA	95 (27.4)
≥3	53 (27.9)	15 (18.1)	38 (35.5)	NA	139 (40.1)
Metastatic sites at palbociclib starting *N* (%)					
Visceral	117 (61.6)	35 (42.2)	82 (76.6)	214 (48.2)	206 (59.4)
Bone only	35 (18.4)	20 (26.5)	15 (14.0)	103 (23.3)	75 (21.6)
Local and/or regional	23 (12.1)	19 (22.9)	4 (3.7)	11 (2.6)	49 (14.1)
Brain	9 (4.7)	0 (0.0)	9 (8.4)	0 (0.0)	NA
Prior chemotherapy for MBC *N* (%)					
Yes	65 (34.2)	0 (0.0)	65 (60.7)	0 (0.0)	113 (32.6)
No	125 (65.8)	83 (100.0)	42 (39.3)	444 (100.0)	234 (67.4)
Concurrent endocrine therapy *N* (%)					
Letrozole	56 (29.5)	33 (39.8)	23 (21.5)	444 (100.0)	0 (0.0)
Anastrozole	21 (11.1)	14 (16.9)	7 (6.5)	0 (0.0)	0 (0.0)
Exemestane	49 (25.8)	18 (21.7)	31 (29.0)	0 (0.0)	0 (0.0)
Fulvestrant	62 (32.6)	18 (21.7)	44 (41.1)	0 (0.0)	347 (100.0)
Tamoxifen	2 (1.1)	0 (0.0)	2 (1.9)	0 (0.0)	0 (0.0)
Treatment line of palbociclib *N* (%)					
1	83 (43.7)	83 (100.0)	0 (0.0)	444 (100.0)	0 (0.0)
2	41 (21.6)	0 (0.0)	41 (38.3)	0 (0.0)	84 (24.2)
≥3	66 (34.7)	0 (0.0)	66 (61.7)	0 (0.0)	263 (75.8)

*Note*: N, number.

Abbreviations: AI, aromatase inhibitor; BMI, body mass index; ECOG, Eastern Cooperative Oncology Group; MBC, metastatic breast cancer; NA, not available.

^a^ Only palbociclib‐combined cohorts.

^b^ Includes patients with newly metastatic disease and patients who developed metastatic disease or recurrence after more than 12 months since completion of prior (neo)adjuvant therapy.

Among patients with recurrent disease, either local or metastatic, approximately 80% received prior neoadjuvant or adjuvant chemotherapy, and 65 patients (34.2%) received prior systemic chemotherapy for MBC. For ET, tamoxifen (56.2%) was the most frequently used adjuvant endocrine agent, followed by aromatase inhibitor (AI) (29.4%), tamoxifen plus gonadotropin‐releasing hormone agonist (GnRHa) (8.5%), and AI plus GnRHa (5.9%). Following the definition of endocrine resistance,^2^ 63.7% of patients resisted last‐line ET (primary resistance: 23.7%; acquired resistance: 40.0%). Comparing our later‐line MBC patients (≥second‐line) with those of the PALOMA‐3 trial, higher proportions of visceral metastasis (76.6% vs. 59.4%) and prior palliative chemotherapy (60.7% vs. 32.6%) were observed in our cohort (Table 1).

### 3.2 Treatment patterns

The median duration on palbociclib treatment was 12.0 months (range: 1–36 months). As shown in Table 1, 43.7% of patients received palbociclib as first‐line treatment (*n* = 83), while 21.6% and 34.7% of patients received palbociclib as second‐line treatment (*n* = 41) or beyond (*n* = 66), respectively. Two‐thirds of the endocrine regimens to be combined with palbociclib were AI (*n* = 126; 66.3%), while 32.6% of patients received fulvestrant (*n* = 62), and 1.1% received tamoxifen (*n* = 2). We explored whether patients' sensitivity to ET would influence doctors' decision to assign endocrine regimens. Among endocrine‐sensitive patients, doctors were more likely to choose nonsteroid AI (letrozole or anastrozole, 47.8%), followed by steroid AI (exemestane) (29.0%) and fulvestrant (21.7%). Among endocrine‐resistant patients, regardless of primary or acquired resistance, fulvestrant was the most commonly chosen regimen (48.9% and 32.9%, respectively) (Figure S1).

For dosing (Table S1), 174 patients (91.6%) were treated at an initial dose of 125 mg/day, of which 20 patients required a dose reduction to 100 mg/day thereafter and 1 eventually reduced to 75 mg/day—a rate (11.1%, *n* = 21) much lower than those in PALOMA‐2 (36.0%) or PALOMA‐3 (34.0%). Most dose reductions (*n* = 19; 90.5%) occurred within the first threecycles. A small proportion of patients were initiated at a lower dose of 100 mg/day (*n* = 15) or 75 mg/day (*n* = 1). Forty‐six patients (24.2%) had at least one cycle delay due to AEs. Only our patients (2.1%) discontinued their treatment due to severe neutropenia.

### 3.3 Efficacy of palbociclib plus ET

The median follow‐up period was 18.0 months (range: 2–36 months). During follow‐up, PFS events were observed in 106 patients (55.8%), and 38 deaths (due to cancer) occurred (20.0%). In general, the 12‐ and 24‐month PFS rates were 53.2% and 38.9%, respectively, and the 12‐ and 24‐month survival rates were 90.3% and 77.1%, respectively.

**FIGURE 1:**
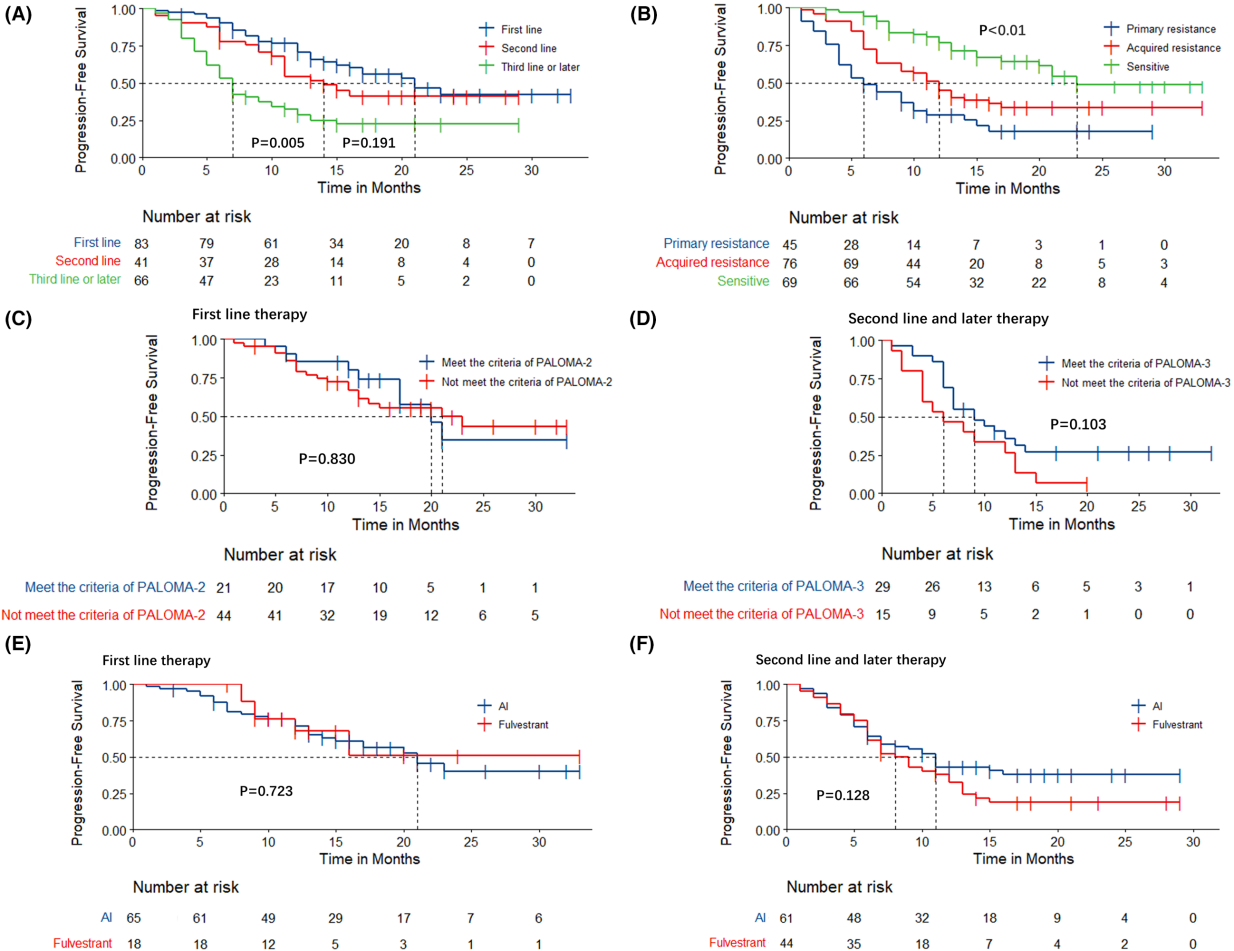
Progression‐free survival for palbociclib plus ET according to patient characteristics stratified by (A) lines of treatment; (B) ET naïve, primary or acquired resistance; (C) whether the inclusion criteria of PALOMA‐2 were met or not; (D) whether the inclusion criteria of PALOMA‐3 were met or not; (E) type of combined ET in first‐line setting; (F) type of combined ET in second‐ and subsequent‐line settings. AI, aromatase inhibitor; ET, endocrine therapy; MBC, metastatic breast cancer

**FIGURE 2:**
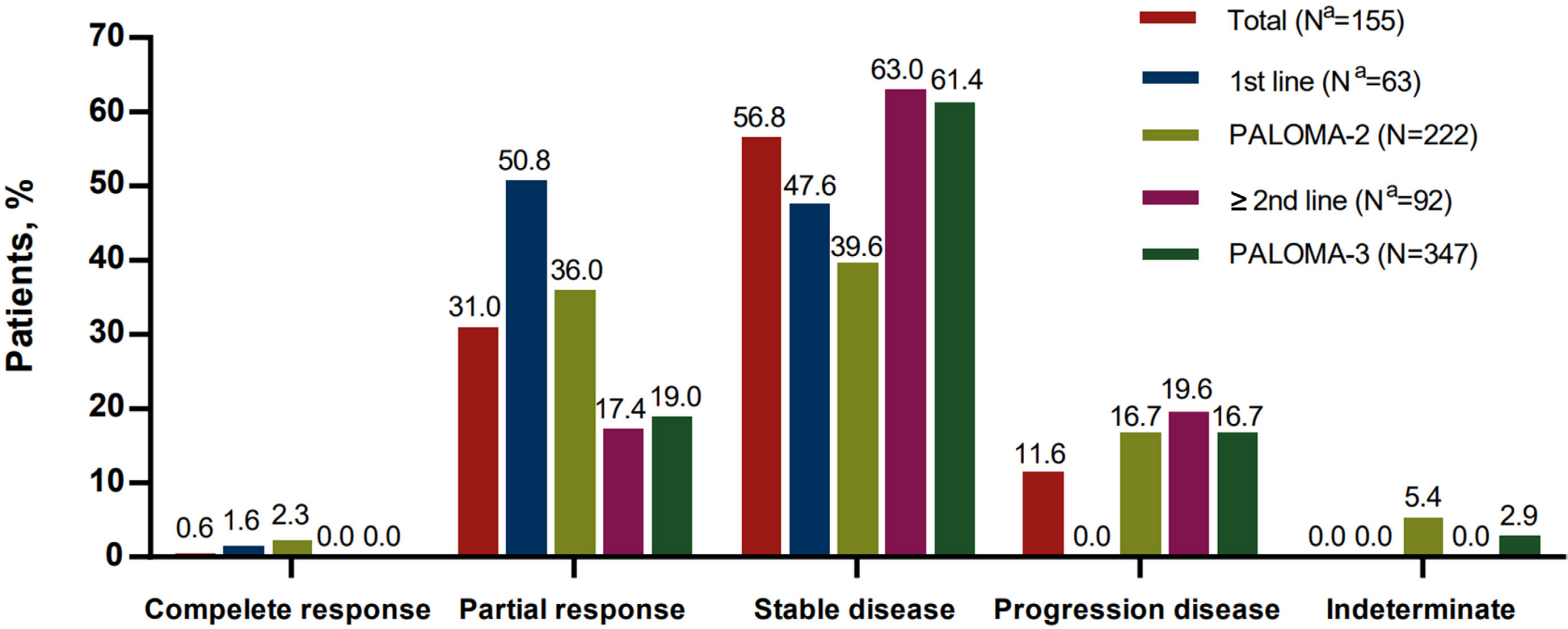
Summary of tumor response to palbociclib treatment in a real‐world population and in the PALOMA‐2/3 population. ^a^Patients with bone‐only metastasis

The mPFS was 21.0 months for patients who received palbociclib as first‐line treatment, in contrast to 14.0 (*p*_log‐rank_ = 0.191) and 7.0 (*p*_log‐rank_ = 0.005) months in second‐ and subsequent‐line settings, respectively (Figure 1A). De novo‐ or endocrine‐sensitive patients showed the longest mPFS of 23.0 months, while patients with acquired and primary resistance showed mPFS values of 12.0 and 6.0 months, respectively (*p*_log‐rank_ <0.01) (Figure 1B). We then assessed the tumor response among patients with measurable disease. The objective response rate (ORR, including complete and partial response) of our first‐line treated patients appeared to be better than that in the PALOMA‐2 trial (52.4% vs. 38.3%), and the ORR of our later‐line treated patients (≥second‐line) was similar to that in the PALOMA‐3 trial (17.4% vs. 19.0%) (Figure 2).

Thereafter, we evaluated the relationships between baseline characteristics and the efficacy of palbociclib plus ET. Patients with visceral metastasis and worse ECOG performance status (≥2) showed significantly poorer survival (*p =* 0.047 and *p =* 0.025), while patients with PR+ and fewer than two metastatic sites presented a favorable prognosis (*p =* 0.027 and *p =* 0.009). As expected, patients who were chemotherapy (HR, 1.75; 95% CI, 1.18–2.58; *p =* 0.005) or ET (HR, 2.47; 95% CI, 1.66–3.67; *p <* 0.001) naïve for metastatic or recurrent disease showed a significantly superior prognosis. No significant difference in PFS was observed according to age (<70 vs. ≥70, *p =* 0.290) or dose reduction (*p =* 0.219) (Figure S2).

We next assessed the prognosis of patients who did not meet the inclusion criteria presented in PALOMA‐2/3. In our first‐line patients (palbociclib plus AI group, *n* = 65), premenopausal status, combined ET (exemestane), and resistance to prior AI were the main reasons for not meeting the inclusion criteria presented in PALOMA‐2, and a comparable mPFS was observed (21.0 vs. 20.0 months, *p*_log‐rank_ = 0.830) (Figure 1C). For patients in our later‐line setting (palbociclib plus fulvestrant group, *n* = 44), the main reasons for being ineligible for PALOMA‐3 were previous treatment with fulvestrant (*n* = 10), poor ECOG performance status (≥2, *n* = 5), and/or receival of more than one prior line of chemotherapy for metastatic disease (*n* = 4). Our results revealed a shorter yet nonsignificant mPFS for those who did not meet the criteria than for those who met the criteria (*n* = 29) (6.0 months vs. 9.0 months, *p*_log‐rank_ = 0.103) (Figure 1D).

The PFS stratified by ET and lines of treatment are listed in Table 2. Patients who received nonsteroidal AI showed similar mPFS to those of patients who received steroidal AI in our first‐line setting (*p*_log‐rank_ = 0.997) as well as in second‐ and later‐line settings (*p*_log‐rank_ = 0.783). When comparing the mPFS of patients administered fulvestrant or AI, a similar efficacy was found regardless of therapeutic lines (Table 2; Figure 1E,F) or of whether patients had previously received AI (fulvestrant vs. AI: 10.0 months vs. 11.0 months, *p*_log‐rank_ = 0.481).

**TABLE 2:** Progression‐free survival by combinations and lines of treatment

Lines of treatment	Combined endocrine therapy	Number of patients	Number of event (progression)	Median PFS (95% CI)	Mean PFS (95% CI)
First	All	83	35	21.0 (14.79–27.21)	21.3 (18.54–24.09)
	Letrozole/anastrozole	47	22	21.0 (15.63–26.37)	20.9 (17.43–24.43)
	Exemestane	18	7	NR	18.2 (13.82–22.59)
	Fulvestrant	18	6	NR	22.6 (15.87–29.25)
Second	All^a^	41	22	14.0 (10.45–17.55)	17.2 (13.80–20.53)
	Letrozole/anastrozole	15	7	NR	18.1 (12.14–24.07)
	Exemestane	14	7	16.0 (8.13–23.87)	16.9 (12.67–21.20)
	Fulvestrant	11	8	11.0 (5.34–16.66)	14.2 (8.46–19.91)
≥Third	All^a^	66	49	7.0 (5.79–8.21)	11.4 (8.87–13.87)
	Letrozole/anastrozole	15	11	7.0 (4.52–9.48)	11.6 (6.17–17.03)
	Exemestane	17	11	6.0 (3.58–8.42)	10.6 (6.81–14.29)
	Fulvestrant	33	26	7.0 (4.79–9.21)	9.6 (7.23–11.98)

Abbreviations: CI, confidence interval; NR, not reached; PFS, progression‐free survival.

^a^ PFS of the only one patient who received tamoxifen was not listed due to limited number of availability.

Regarding regimens administered after disease progression on palbociclib, 80 of 106 patients were documented to receive other treatments, including 56 patients switched to chemotherapy, 15 patients continuing ET (mostly fulvestrant), six patients prescribed everolimus, and three patients prescribed chidamide. Twenty‐four patients chose to forego therapy due to pejorative general condition or poor economic condition. Two patients were lost to follow‐up.

### 3.4 Adverse events

As shown in Table 3, the most reported AE in our population was neutropenia (74.2%), and grade 3/4 neutropenia was observed in 57 of 190 (30.0%) patients. Other hematological AEs, such as thrombocytopenia (22.1%) and anemia (32.6%), were documented more frequently in our cohort than in the PALOMA‐2/3 trials, yet rarely any grade 3/4 AEs were reported. Fatigue was the most reported nonhematological AE, followed by decreased appetite and stomatitis. No significant difference in AEs by lines of palbociclib treatment was observed. The main reason underlying dose adjustment was hematological toxicity, including neutropenia, leukopenia, and thrombocytopenia.

**TABLE 3:** Treatment‐related adverse events in our study and the PALOMA‐2/3 trials

	Total (*N =* 190)	First line (*N =* 83)	≥Second line (*N =* 107)	PALOMA‐2 (*N =* 444)	PALOMA‐3 (*N =* 345)
Neutropenia *N* (%)	141 (74.2)	62 (74.7)	79 (73.8)	353 (79.5)	279 (80.9)
Grade 3/4 neutropenia *N* (%)	57 (30.0)	21 (25.3)	36 (33.6)	295 (66.5)	223 (64.6)
Febrile neutropenia *N* (%)	8 (4.2)	2 (2.4)	6 (5.6)	8 (1.8)	3 (0.9)
Thrombocytopenia *N* (%)	42 (22.1)	19 (22.9)	23 (21.5)	69 (15.5)	73 (21.2)
Anemia *N* (%)	62 (32.6)	25 (30.1)	37 (34.6)	107 (24.1)	96 (27.8)
Fatigue *N* (%)	92 (48.4)	35 (42.2)	57 (53.3)	166 (37.4)	135 (39.1)
Nausea *N* (%)	15 (7.9)	6 (7.2)	9 (8.4)	156 (35.1)	112 (32.5)
Stomatitis *N* (%)	27 (14.2)	12 (14.5)	15 (14.0)	68 (15.3)	43 (12.5)
Rash *N* (%)	14 (7.4)	9 (10.8)	5 (4.7)	79 (17.8)	52 (15.1)
Alopecia *N* (%)	10 (5.3)	4 (4.8)	6 (5.6)	146 (32.9)	58 (16.8)
Diarrhea *N* (%)	15 (7.9)	6 (7.2)	9 (8.4)	116 (26.1)	74 (21.4)
Decreased appetite *N* (%)	31 (16.3)	12 (14.5)	19 (17.8)	66 (14.9)	52 (15.1)

*Note*: N, number.

### 3.5 Patient‐reported outcomes

Eighty‐two patients completed both the EORTC QLQ‐C30 and EQ‐5D questionnaires. The median duration of treatment for these patients was 10.9 months (95% CI, 4.8–17.0).

As shown in Table S2, the overall global quality of life (QoL) scores of the QLQ‐C30 were significantly improved posttreatment [75.8 (95% CI, 74.2–77.4) vs. 88.4 (95% CI, 87.2–89.6), *p* < 0.0001]. For functioning scales, the mean scores of physical, role, and social function scales remained unchanged, while significant improvement was observed in cognitive [93.5 (95% CI, 91.9–95.1) vs. 96.5 (95% CI, 95.5–97.5), *p* = 0.018] and emotional [89.9 (95% CI, 88.1–91.7) vs. 94.5 (95% CI, 93.6–95.4), *p* = 0.011] function at posttreatment (Figure S3). For symptom scales, except for pain, which was significantly relieved [14.1 (95% CI, 12.1–16.1) vs. 6.5 (95% CI, 5.0–8.0), *p* < 0.0001)], the other symptoms remained similar at baseline and posttreatment. As expected, palbociclib treatment caused a negative financial impact on patients [19.9 (95% CI, 16.8–21.6) vs. 50.1 (95% CI, 46.0–54.2), *p* < 0.0001].

Regarding the EQ‐5D, the proportion of patients reporting “some problems” in pain/discomfort and anxiety/depression was significantly reduced posttreatment (43.9% vs. 29.3%, *p* < 0.0001; 39.0% vs. 24.4%, *p* < 0.0001) (Table S3). Significant improvement was also observed at posttreatment for the EQ‐5D index score [0.653 (95% CI, 0.622–0.684) vs. 0.819 (95% CI, 0.777–0.861), *p* < 0.0001] and visual analog scale (VAS) [65.1 (95% CI, 58.5–71.7) vs. 78.2 (95% CI, 72.1–84.3), *p* < 0.0001].

### 3.6 Meta‐analysis of published RWSs

Eleven articles (10 retrospective studies and 1 prospective study) satisfying the selection criteria were included in our meta‐analysis (Figure S4). Briefly, a total of 2699 patients were included, with 1601 patients receiving palbociclib plus ET as first‐line treatment, 325 patients as second‐line treatment, and 773 patients as third‐line treatment or beyond. The median follow‐up time ranged from 7.6 to 24.2 months. Details of the patient characteristics in each study are listed in Table S4.

In both first‐ and second‐line settings, the estimated mPFS was roughly consistent with that of our cohort (first line: 18.65 months vs. 21.0 months; second line: 10.61 months vs. 14.0 months) (Figure S5). While in the third‐line setting or beyond, the meta‐analyzed mPFS was shorter than that in our cohort (4.81 months vs. 7.0 months), possibly due to the inclusion of three compassionate programs^18^, ^19^, ^20^ involving heavily pretreated and endocrine‐resistant patients. No obvious publication bias was observed (Figure S6).

## 4 DISCUSSION

CDK4/6i plus ET has become the first choice for HR+HER2− ABC patients based on the results from a series of randomized controlled trials (RCTs), in which both PFS and OS were shown to be greatly improved.^9^, ^21^, ^22^ Nevertheless, the underrepresentation of the Chinese population as well as the highly controlled environment of RCTs make it difficult to precisely evaluate the efficacy and safety of palbociclib in real‐world China, especially among patients with complicated conditions. To the best of our knowledge, this is the largest multicenter study hitherto conducted in a Chinese patient population addressing the real‐world efficacy and patterns of palbociclib treatment, the only CDK4/6i available in China until April 2021.

Our study has several important clinical implications. First, we found an inferior yet nonsignificant prognosis for patients who failed to meet the inclusion criteria of PALOMA‐3 than those who met the criteria (later‐lines: 6.0 months vs. 9.0 months), suggesting that more advanced cancer or pretreatment, which were features of the ineligible subpopulation, are likely to attenuate the efficacy of palbociclib. Second, it is worth noting that an extremely poor prognosis (mPFS: 6.0 months) was observed in patients with poor ECOG performance status (≥2) regardless of prior chemotherapy for MBC, indicating the importance of evaluating patient baseline status before administrating palbociclib. Moreover, although both RCTs^7^, ^8^ and integrated meta‐analyses^23^ have confirmed the priority of CDK4/6i in the first‐ and second‐line treatment for HR+HER2− MBC, especially for those who are chemotherapy naïve and endocrine sensitive, it remains controversial whether CDK4/6i is superior to chemotherapy in patients who have received chemotherapy for MBC or are endocrine resistant. Indeed, the recently published PEARL trial did not find an improvement in PFS when using the palbociclib‐ET combination compared with capecitabine in endocrine‐resistant MBC patients (mPFS: 8.0 months vs. 10.6 months).^24^ Such findings are possibly due to the inclusion of a greater proportion of heavily pretreated patients (41.2% had more than second‐line treatment, 69.3% were AI resistant and 28.8% were fulvestrant resistant). Consistently, in our cohort, heavily pretreated patients (≥third‐line, accounting for 34.7%) also exhibited a comparably poor mPFS of 7.0 months. Perhaps, not surprisingly, certain chemotherapies remain optimal choices for these patients. For example, the efficacy of eribulin in third‐ or fourth‐line treatment for MBC has been investigated and proven to produce a survival benefit over other chemotherapies, including capecitabine, vinca alcaloids, and taxanes and especially in HER2‐ patients,^25^ and the mPFS of eribulin in heavily pretreated HR+HER2− patients was 4.1 months.^26^ Therefore, unlike with chemotherapy, the survival benefit of CDK4/6i remains undetermined for heavily pretreated patients (prior multiple‐line therapy and/or endocrine resistance). Considering its high price in China, careful evaluation of the treatment options, particularly for those patients, is needed.

The latest published results of PALOMA‐3 in ASCO (2021) revealed a significantly improved OS in the palbociclib‐treated group but not a significant benefit for patients with primary resistance,^21^ in line with results from other RWSs.^27^, ^28^ However, another CDK4/6i, abemaciclib, presented a similar benefit between patients with primary and secondary endocrine resistance (16.3 months vs. 16.9 months) in MONARCH‐2.^9^ Such a discrepancy could be partly due to the differences in patient populations: more than one line of ET or prior chemotherapy for MBC was allowed in PALOMA‐3 but was restricted in MONARCH‐2. In our cohort, 60% of patients (27/45) with primary endocrine resistance also received chemotherapy and/or more than one line of ET for MBC. As expected, their mPFS was much shorter than that of their counterparts who exhibited primary endocrine resistance but were chemotherapy naïve and who were administered less than two lines of ET for MBC (5.0 months vs. 9.0 months). Furthermore, a phase II trial, KCSG‐BR15‐10, showed that palbociclib plus exemestane and GnRHa produced a significantly longer PFS than that achieved with capecitabine in premenopausal patients (*p* = 0.024), while the benefit was uncertain for chemotherapy pretreated patients (HR, 0.82; 95% CI, 0.36–1.92).^29^ These results collectively suggest that palbociclib should be used with caution among those who are primary endocrine resistant and have received chemotherapy for MBC. Additional evidence is needed to determine whether abemaciclib benefits these patients.

The optimal ET partner (AI or fulvestrant) for palbociclib remains to be identified. To assess the superiority of letrozole and fulvestrant in endocrine‐sensitive HR+HER2− MBC, PARSIFAL was conducted and revealed a similar PFS and OS between the two groups of patients administered each regimen.^30^ Despite being nonsignificant, the subgroup analysis showed a longer mPFS with fulvestrant than letrozole for patients who received prior AI therapy (27.5 months vs. 19.3 months), which is similar to our result irrespective of the treatment lines or previous ET regimen. ESR1 mutation is known as one of the most crucial mechanisms of ET resistance in advanced HR+ BC, and the BOLERO‐2 trial revealed that 28.8% of AI‐treated BC patients could have ESR1 mutations.^31^ In addition, previous clinical trials (EFECT and SoFEA) indicated a better efficacy of fulvestrant than for exemestane in HR+ BC patients who progressed on nonsteroid AI treatment.^32^, ^33^ Recently, several trials also implied that ESR1 mutation status should also be considered when choosing the partner of CDK4/6i. The PEARL trial found that the efficacy of palbociclib plus exemestane was much worse in patients with baseline ESR1 mutations (mPFS: mutations, 5.7 months; wild‐type, 9.3 months) but yielded a similar PFS for patients on palbociclib plus fulvestrant (mPFS: 7.5–7.6 months).^24^ Moreover, PALOMA‐3 also confirmed that the efficacy of palbociclib plus fulvestrant was not influenced by ESR1 mutation status.^34^ The PADA‐1 trial first explored the association between baseline ESR1 status and its dynamic change in circulating free DNA (cfDNA) with the treatment efficacy of palbociclib plus AI,^35^ and the first‐stage result released in ASCO 2020 confirmed that the baseline ESR1 mutation was a prognostic marker for patients treated with palbociclib and AI (mPFS: 11.0 months vs. 26.7 months).^36^ Moreover, ESR1 mutation clearance in cfDNA was observed in 70% of patients, which indicated that AI plus palbociclib still retained some activity irrespective of ESR1 mutation.^36^ Altogether, these findings suggested that ESR1 mutation screening should be considered before first‐line therapy with palbociclib plus AI, especially in patients who had AI prior to adjuvant ET. In addition, a diversity of combined ET in different treatment lines was found in our cohort: unlike in PALOMA‐2/3,^7^, ^8^ only 56.6% of patients in the first‐line setting received nonsteroid AI, while 58.9% of patients in the later‐line setting did not receive fulvestrant as combined ET, suggesting that patients' medication history does not appear to influence physicians' choices in the real world. This finding was supported by other RWSs, including the Ibrance Real World Insights (IRIS) study in the United States^37^ and a Chinese retrospective study conducted by Liu et al.,^38^ highlighting a diversity of combined ET in different treatment lines.

The safety profile was comprehensively documented by electronic medical records in our study. Similar to PALOMA‐2/3, the most common AE in our cohort was neutropenia (74.2%), while the proportion of patients with grade 3/4 neutropenia (30.0%) was much lower than those in PALOMA‐2 (66.5%) and PALOMA‐3 (64.6%).^7^, ^8^ Additionally, a prospective registry study in Germany found a much lower rate of neutropenia in the palbociclib group (11.3%; grade 3/4: 3.5%),^39^ and another study involving Latin Americans observed a mere 20% of patients with grade 3/4 neutropenia.^40^ It is well‐known that the AEs reported in RWSs are often different from those reported in RCTs.^41^ In our cohort, 32.1% of patients with neutropenia received oral administration of leucogen tablets to protect bone marrow suppression, a possible explanation for the low observed proportion of grade 3/4 neutropenia events. In addition, a lower rate of nausea, rash, alopecia, and diarrhea was found in our study than with RCTs, while a higher rate of anemia and thrombocytopenia was observed in our study, yet rarely any grade 3/4 AEs were reported, demonstrating a good safety of palbociclib in the Chinese population.

Given that almost all patients eventually develop tumor progression on CDK4/6i treatment, which results in a heavy economic burden, it is critical to understand patients' QoL in addition to efficacy. Attenuation of anxiety, depression, and pain symptoms is known to be associated with favorable cancer‐specific survival in BC patients.^42^, ^43^ In our benefit–risk assessment results, palbociclib plus ET significantly improved overall QoL, cognitive, and emotional functioning (including anxiety and depression), and pain symptoms relative to baseline levels in a real‐world Chinese population, consistent with the results of PROs in PALOMA‐2/3.^44^, ^45^

Several limitations need to be acknowledged. First, although this is the RWS with the largest sample size and longest follow‐up duration to date to focus on Asian MBC patients (Figure S7), our sample size is still considered small. We look forward to larger RWSs to be conducted in the future with joint efforts from an enlarged number of participating centers. Second, it is likely that physicians prefer to prescribe certain concurrent ETs over other ETs, which might influence observed outcomes. We hope that our multicenter design could to some extent randomize such bias and reduce its impact to a minimal extent. Finally, potential recall bias in QoL questionnaires for baseline status is difficult to avoid.

## 5 CONCLUSIONS

Our study confirmed the efficacy and tolerability of palbociclib combined with ET in treating Chinese HR+HER2− MBC. Moreover, palbociclib significantly improved the QoL of our patients. The differences in AEs among Chinese patients need to be explored and confirmed by prospective controlled clinical trials in the future.

## CONFLICT OF INTEREST

The authors declare that they have no conflicts of interest.

## AUTHOR CONTRIBUTIONS

Chao Ni, Xia Jiang, Jian Huang, and Hengyu Li designed and supervised the study. Chao Ni, Lesang Shen, Jun Zhou, and Yiding Chen wrote the manuscript. Xia Jiang, Lesang Shen, and Jun Zhou performed data analysis. Jinhua Ding, Haiyan Wei, Jian Liu, Wenjie Xia, Bojian Xie, Xiaohong Xie, Xujun Li, Yuechu Dai, Guobing Zhang, Xia Qiu, Chao Li, Shanshan Sun, Wuzhen Chen, and Dihe Gong contributed to patient recruitment, data collection, and patient follow‐up. All authors reviewed and approved the final manuscript.

## ETHICAL APPROVAL STATEMENT

This study was approved by the ethics committees of each participating hospital and was conducted in accordance with the Declaration of Helsinki.

## Supporting information


Data S1

## Data Availability

All relevant data are available in the supplementary files or upon request from the corresponding author.
