# Supplementary material for: Treatment patterns, effectiveness, and patient‐reported outcomes of palbociclib therapy in Chinese patients with advanced breast cancer: A multicenter ambispective real‐world study
Source: Cancer Med. 2022 Apr 25;11(22):4157–68. doi: 10.1002/cam4.4767 (PMC9678098; doi:10.1002/cam4.4767)
Supplement: Supplementary file 1 — Data S1 [file CAM4-11-4157-s001.docx]

Supplementary Material

# Supplementary data

## Name of participating hospitals

The Second Affiliated Hospital of Zhejiang University,

The First Affiliated Hospital of Zhejiang University,

Taizhou Enze Medical Center Enze Hospital,

Affiliated Hangzhou First People’s Hospital of Zhejiang University,

Affiliated Changhai Hospital of The Second Military Medical University,

Hwa Mei Hospital University of Chinese Academy of Sciences,

Affiliated Hangzhou Cancer Hospital of Zhejiang University,

Zhejiang Provincial People’s Hospital,

The First Affiliated Hospital of Zhejiang Chinese Medical University,

Zhoushan Hospital of Zhejiang University,

Taizhou Municipal Hospital,

Ningbo Medical Center Lihuili Hospital.

## Assessment of patient-reported outcomes

**The EORTC QLQ-C30 questionnaire:** The EORTC QLQ-C30 was a 30-item questionnaire composed of a global QoL subscale, five multi-item functional subscales (physical, role, emotional, cognitive, and social functioning), three multi-item symptom scales (fatigue, nausea/vomiting, and pain), and five single-item symptom scales assessing other cancer-related symptoms.

**The EQ-5D questionnaire:** The EQ-5D questionnaire consisted of a five-item health status measure and a visual analog scale (VAS) administered separately. Two scores (the EQ-5D index score and the VAS score) were generated from this questionnaire. The EQ-5D index score was resulted from answers to the five-item questionnaire evaluating mobility, self-care, usual activities, pain, discomfort, and anxiety/depression. The scores of answers depended on whether patients noticed no problems (=1), some problems (=2), or extreme problems (=3). The EQ-5D index scores were calculated using an algorithm based on societal preferences from the general population-based valuation studies in China (0=death, 1=perfect health). The EQ-5D VAS was ranged from 0 (worst health status) to 100 (best health status) rating their current health.

## Systematic review of real-world studies

**Literature screening:** All publications written in English as expanded access programme (EAP), compassionate programme, retrospective or prospective studies were collected and their titles and abstracts were reviewed by two investigators (LSS and JZ) independently. Full-text analysis and data extraction were carried out independently by two researchers (LSS and XJ). A quality check against source material was conducted by CN.

**Inclusion and exclusion criteria of meta-analysis:** Studies selected for the final analysis met the following criteria: clinical studies on breast cancer patients; studies using palbociclib as a combination therapy; be observational (not randomized or intervention); progression-free survival outcome was reported. Exclusion criteria included studies not in full-length (conference abstracts); studies with less than 50 patients; repeated publications or reports of the same study; or biomarkers studies, economic analysis, quality of life evaluation, and other studies not reporting real-world data.

**Analytical approaches:** Study characteristics including year of publication, research type, country, sample size, and other demographic and clinicopathological features of patients were extracted. Outcome median PFS with its 95% CIs was extracted. Heterogeneity was assessed using the Cochrane Q statistic (significant heterogeneity if P<0.10) and the Higgins’ I2 statistic (significant if >50%). Both random and fixed effects models were presented, while in the text, we showed the results derived from the random effect model to account for heterogeneity. Publication bias was assessed via funnel plots. All statistical analysis were performed using R software version 4.0.3.

# Supplementary Figures and Tables

## Supplementary Figures


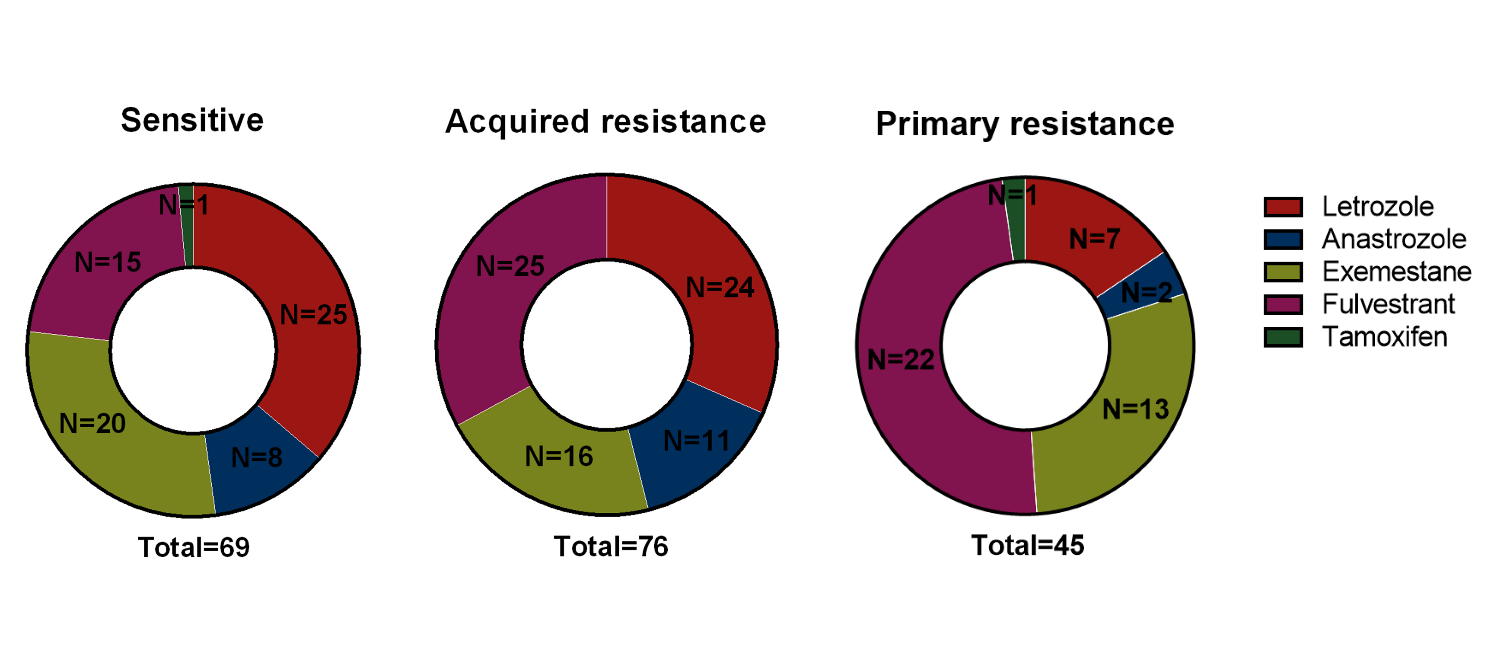


**Supplementary Figure 1.** The choice of combined endocrine regimen in patients with different endocrine sensitivities.


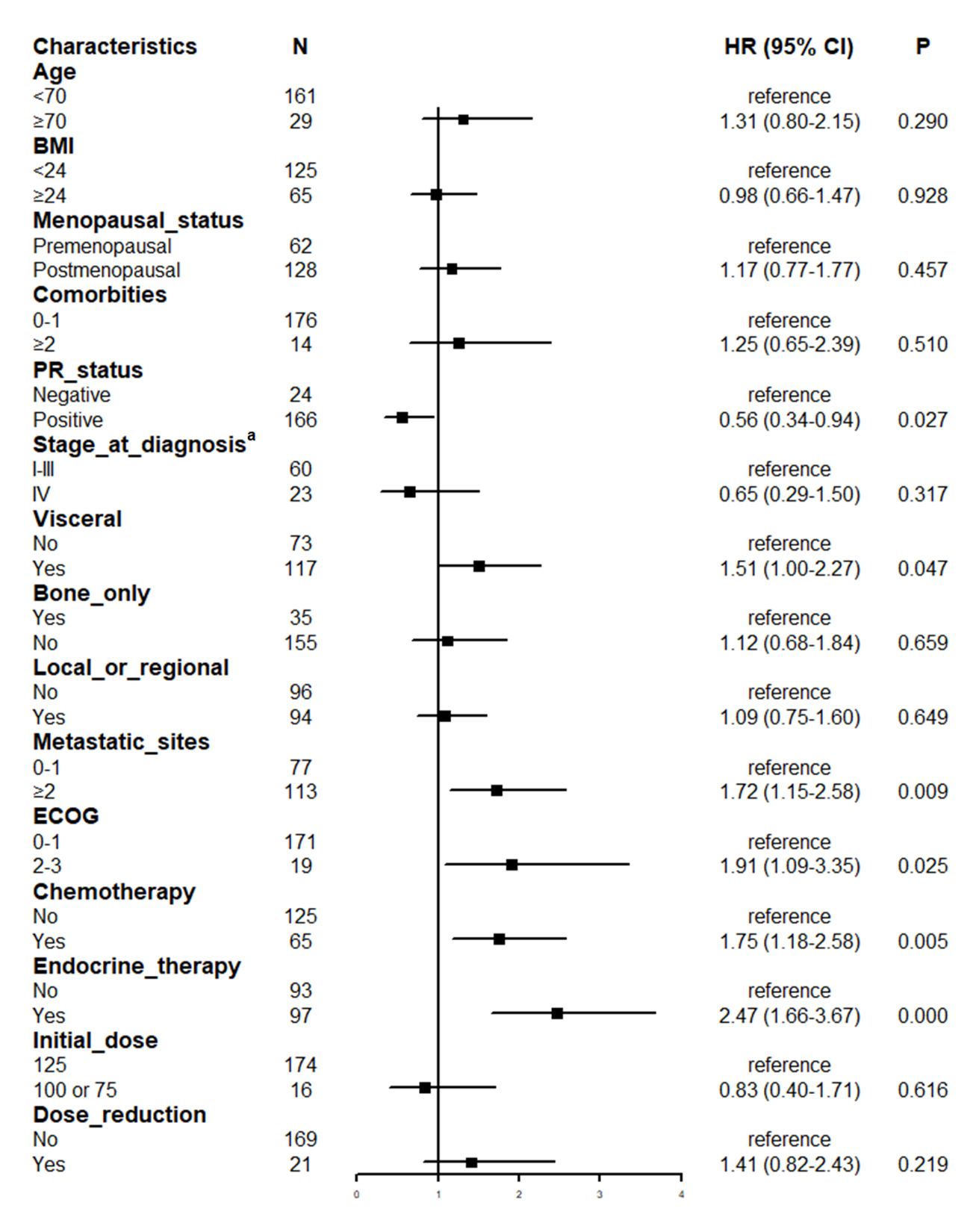


**Supplementary Figure 2.** Subgroup analysis of progression-free survival.

Abbreviation: BMI, body mass index; CI, confidence interval; ECOG, Eastern Cooperative Oncology Group; HR, hazard ratio; N, number; PR, progesterone receptor.

^a^ Patients who received palbociclib as first-line treatment.


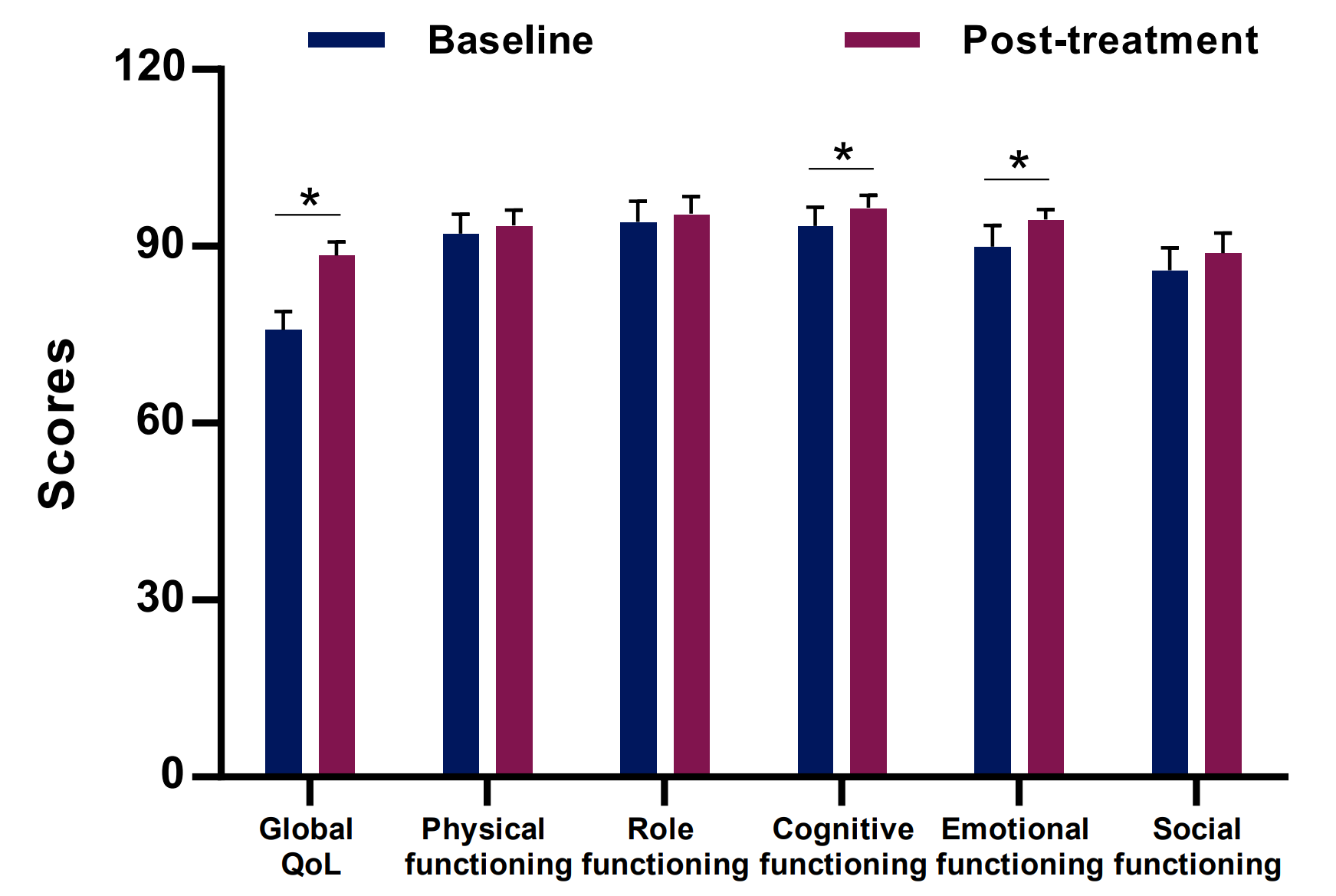


**Supplementary Figure 3.** The change in EORTC QLQ-C30 scores of global QoL and functional scales at baseline and posttreatment. The scores are presented as mean and 95% CI.

*P<0.05. Abbreviation: EORTC QLQ-C30, European Organization for Research and Treatment of Cancer Quality of Life Questionnaire-Core 30 items; QoL, quality of life.


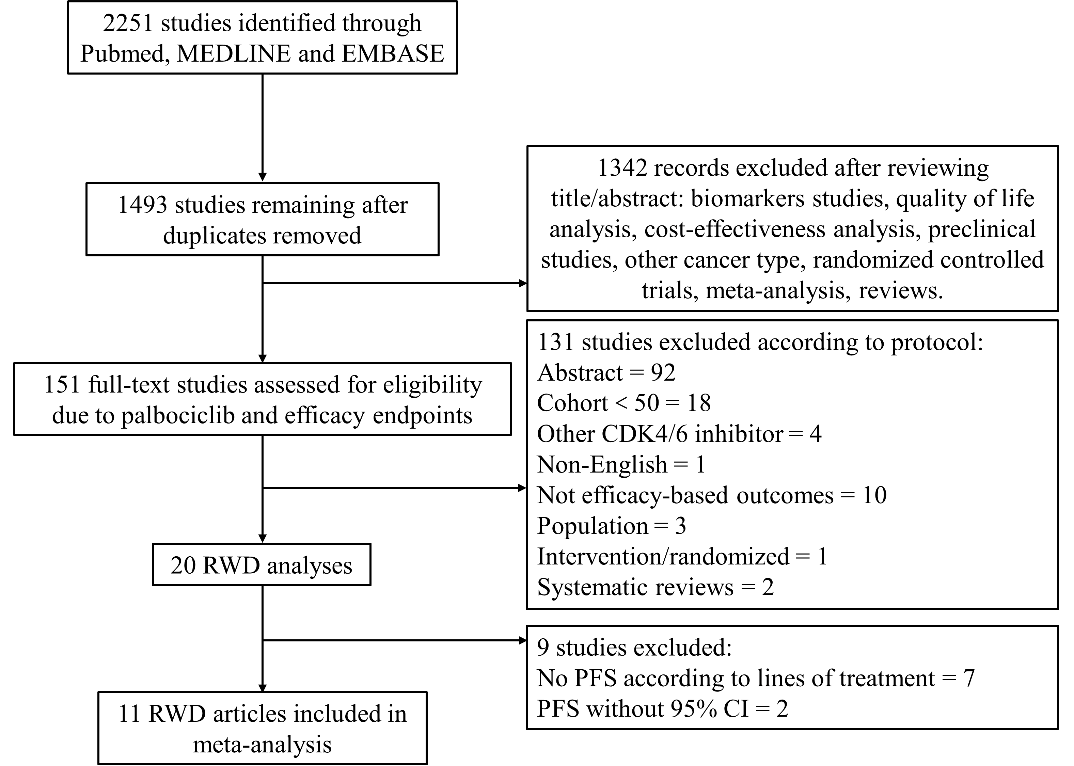


**Supplementary Figure 4.** PRISMA flow diagram of the literature search and study selection.

Abbreviation: CDK, cyclin-dependent kinases; CI, confidence interval; PFS, progression-free survival; PRISMA Preferred Reporting Items for Systematic Reviews and Meta-Analyses; RWD, real-world data.


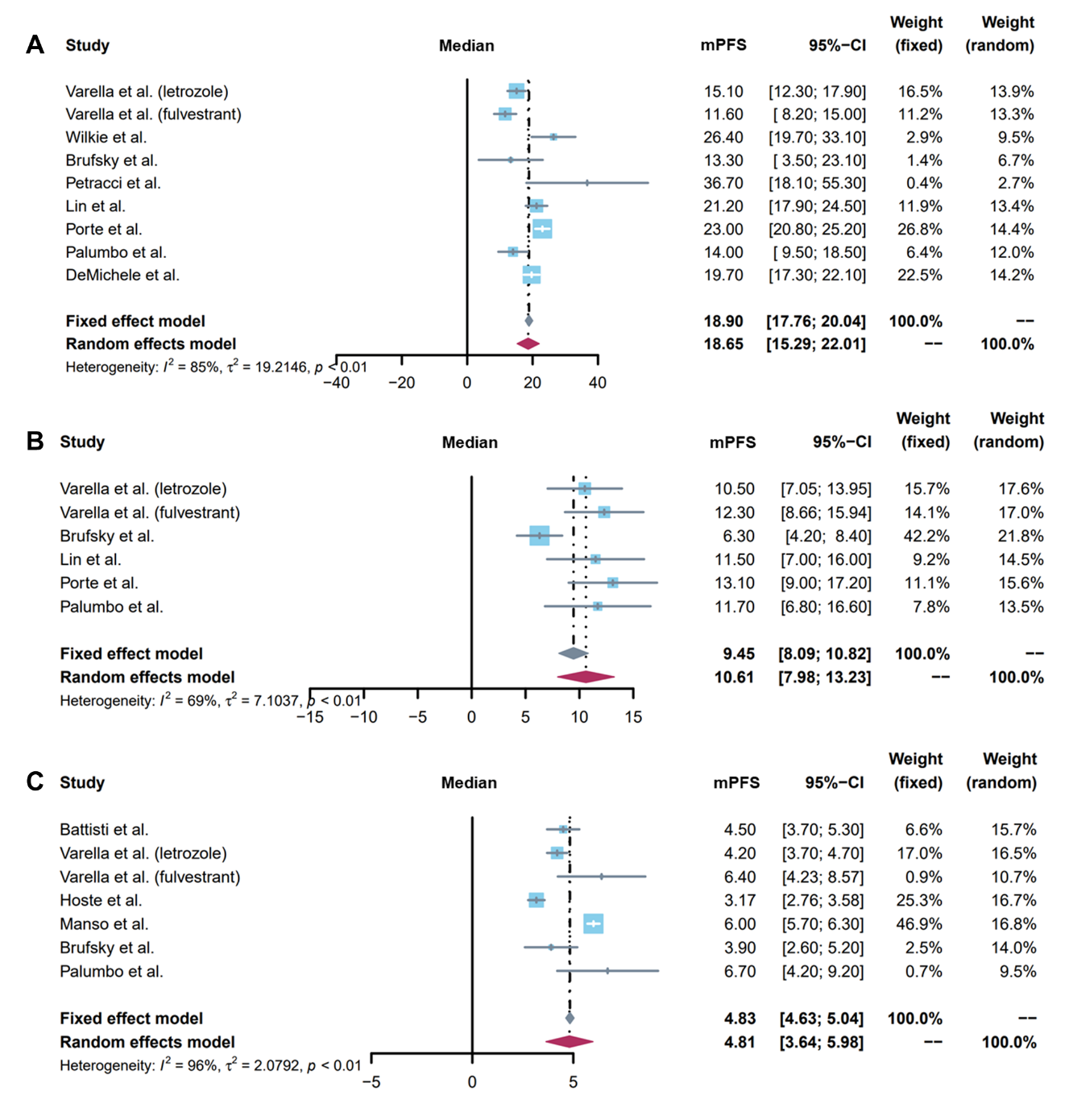


**Supplementary Figure 5.** Forest plot of mPFS for patients treated with palbociclib in the (A) first-line setting, (B) second-line setting, and (C) third-line setting and beyond.

Abbreviation: CI, confidence interval; mPFS, median progression-free survival.


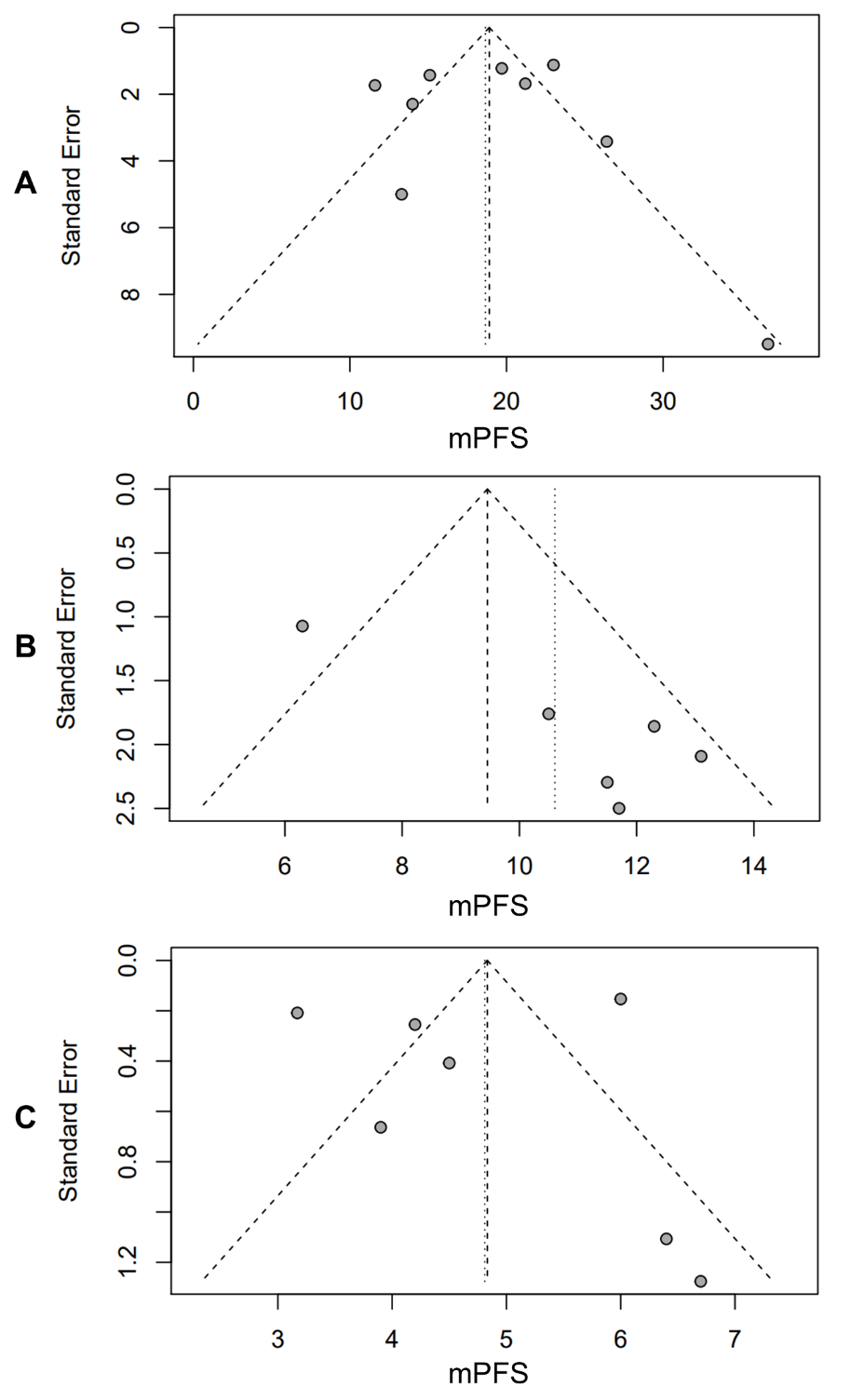


**Supplementary Figure 6.** Funnel plot to detect publication bias. (A) First-line setting; (B) second-line setting; (C) third-line setting and beyond.

Abbreviation: mPFS, median progression-free survival.


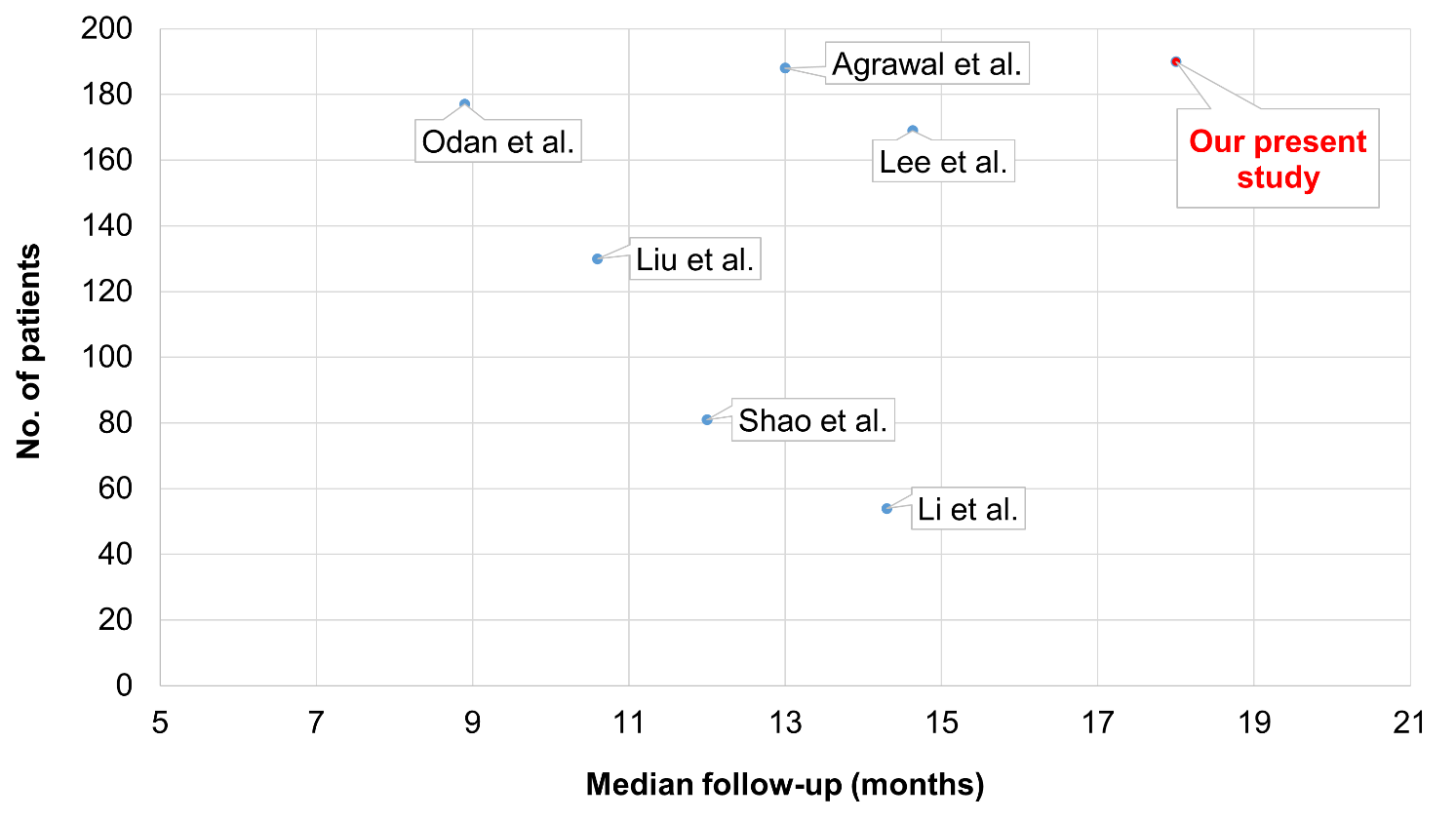


**Supplementary Figure S7.** Published retrospective cohorts with palbociclib in Asia.

## Supplementary Tables

| **Supplementary Table S1.** Dose adjustments of palbociclib in our patient population. | |
| --- | --- |
|  | **N=190** |
| **Starting dose (mg/day) N (%)** |  |
| 125 | 174 (91.6) |
| 100 | 15 (7.9) |
| 75 | 1 (0.5) |
| **Dose reduction (mg/day) N (%)^a^** |  |
| From 125 to 100 | 20 (95.2) |
| From 125 to 75 | 1 (4.8) |
| **Cycle of dose reduction N (%)^a^** |  |
| 2 | 12 (57.1) |
| 3 | 7 (33.3) |
| ≥4^b^ | 2 (9.5) |
| **Dsicontinuation N (%)** |  |
| Yes | 4 (2.1) |
| No | 186 (97.9) |
| **Cycle delay N (%)** |  |
| Yes | 46 (24.2) |
| No | 144 (75.8) |
| **Reason for dose adjustment N (%)^a^** |  |
| Neutropenia | 19 (90.5) |
| Thrombocytopenia | 1 (4.8) |
| Leukopenia | 1 (4.8) |
| **mPFS of patients who received dose reduction (95% CI), months** | 9.0 (0.0-18.0) |
| **mPFS of patients who did not receive dose reduction (95% CI), months** | 13.0 (9.9-16.1) |
| Abbreviation: CI, confidence interval; mPFS, median progression-free survival; N, number.  ^a^ Percentage among patients who received dose reduction (N=21).  ^b^ Cycle 4 and cycle 7, respectively. | |

| **Supplementary Table S2.** Summary of EORTC QLQ-C30 scores at times of baseline and post-treatment. | | | | | |
| --- | --- | --- | --- | --- | --- |
| Domain/scale | Items | N^a^ | Before treatment | After treatment | *P* |
| EORTC QLQ-C30 |  |  | Mean (95% CI) | Mean (95% CI) |  |
| **Global QoL and Functioning scales** | | | | | |
| Physical | 1-5 | 82 | 92.2 (90.5-93.9) | 93.6 (92.3-94.9) | 0.132 |
| Role | 6.7 | 82 | 94.1 (92.3-95.9) | 95.5 (94.1-96.9) | 0.231 |
| Cognitive | 20.25 | 82 | 93.5 (91.9-95.1) | 96.5 (95.5-97.5) | 0.018 |
| Emotional | 21-24 | 82 | 89.9 (88.1-91.7) | 94.5 (93.6-95.4) | 0.011 |
| Social | 26.27 | 82 | 86.0 (84.1-87.9) | 88.8 (87.1-90.5) | 0.253 |
| Global quality of life | 29.30 | 82 | 75.8 (74.2-77.4) | 88.4 (87.2-89.6) | <0.0001 |
| **Symptom scales** | | | | | |
| Fatigue | 10.12.18 | 82 | 9.6 (7.6-11.6) | 8.6 (7.2-10.0) | 0.574 |
| Nausea and vomiting | 14.15 | 82 | 1.5 (0.6-2.4) | 1.1 (0.2-2.0) | 0.654 |
| Pain | 9.19 | 82 | 14.1 (12.1-16.1) | 6.5 (5.0-8.0) | <0.0001 |
| Dyspnea | 8 | 82 | 4.9 (3.2-6.6) | 2.6 (1.4-3.8) | 0.112 |
| Sleep disturbance | 11 | 82 | 9.6 (7.3-11.9) | 8.8 (6.6-11.0) | 0.411 |
| Appetite loss | 13 | 82 | 4.3 (2.5-6.1) | 4.0 (2.4-5.6) | 0.675 |
| Constipation | 16 | 82 | 6.9 (5.3-8.5) | 4.5 (2.5-6.4) | 0.225 |
| Diarrhea | 17 | 82 | 2.0 (1.0-3.0) | 1.1 (0.3-1.9) | 0.129 |
| Financial impact | 28 | 82 | 19.9 (16.8-21.6) | 50.1 (46.0-54.2) | <0.0001 |
| Abbreviation: C30, core 30 items; CI, confidence interval; EORTC QLQ, European Organization for Research and Treatment of Cancer Quality of Life Questionnaire; N, number.  ^a^ Number of patients with data available for the corresponding visit. | | | | | |

| **Supplementary Table S3.** Summary of EQ-5D scores at baseline and post-treatment. | | | | | | | | |
| --- | --- | --- | --- | --- | --- | --- | --- | --- |
| Health state | N | **Before treatment** | | | **After treatment** | | | *P* |
|  |  | No problem N (%) | Some problem N (%) | Extreme problem N (%) | No problem N (%) | Some problem N (%) | Extreme problem N (%) |  |
| Mobility | 82 | 67 (81.7) | 15 (18.3) | 0 | 69 (84.1) | 13 (15.9) | 0 | 0.301 |
| Self-care | 82 | 72 (87.8) | 10 (12.2) | 0 | 73 (89.0) | 9 (11.0) | 0 | 0.698 |
| Usual activities | 82 | 68 (82.9) | 14 (17.1) | 0 | 70 (85.4) | 12 (14.6) | 0 | 0.159 |
| Pain/discomfort | 82 | 46 (56.1) | 36 (43.9) | 0 | 58 (70.7) | 24 (29.3) | 0 | ＜0.0001 |
| Anxiety/depression | 82 | 50 (61.0) | 32 (39.0) | 0 | 62 (75.6) | 20 (24.4) | 0 | ＜0.0001 |
|  |  | **Mean (95% CI)** | | | **Mean (95% CI)** | | |  |
| **EQ-5D index score^a^** | 82 | 0.653 (0.622-0.684) | | | 0.819 (0.777-0.861) | | | ＜0.0001 |
| **EQ-5D VAS^a^** | 82 | 65.1 (58.5-71.7) | | | 78.2 (72.1-84.3) | | | ＜0.0001 |
| Abbreviation: CI, conﬁdence interval; EQ-5D, EuroQOL 5 dimensions questionnaire; *N*, number; VAS, visual analog scale.  ^a^ Higher EQ-5D index and VAS scores indicate better health status/quality of life. | | | | | | | | |
|  | | | | | | | | |

| **Supplementary Table S4.** Main characteristics of published articles included in our meta-analysis. | | | | | | | | | | | |
| --- | --- | --- | --- | --- | --- | --- | --- | --- | --- | --- | --- |
| Year of publication | 2018(1) | 2019(2) | 2019(3) | 2019(4) | 2019(5) | 2020(6) | 2020(7) | 2020(8) | 2020(9) | 2021(10) | 2021(11) |
| First author’s name | Hoste | Wilkie | Varella | Battisti | Brufsky | Petracci | Lin | Manso | Porte | Palumbo | DeMichele |
| Multi-centers or not | No | No | No | Yes | Yes | Yes | Yes | Yes | No | Yes | Yes |
| Research type | Compassion Programme | University hospital | Not university hospital | Compassion Programme | EAP | Not university hospital | Database | Compassion Programme | Not university hospital | University hospital (prospective) | Database |
| Country | Belgium | America | America | UK | America | Argentina | America | Spain | France | Italy | America |
| Median follow-up (months) | Unknown | Unknown | 10.2 | Unknown | 14.2 | Unknown | 7.6-10.8 | Unknown | 20.7 | 24 | 24.2 |
| Patient ethnicity | Unknown | 88% white  9% black | 86% white  12% black | Unknown | 83% white  9% black | Latin American | 75% white  20% black | Unknown | Unknown | Unknown | 68% white  7% black |
| Median age (years) | 67.1 | 64 | 53.5 | 59 | 62.5 | 57 | 64.5 | 58 | 61.8 | 62 | 66 |
| Patient age range (years) | 34.8-85.9 | 31-88 | 17-83 | 32-82 | 37-89 | 29-84 | 30.6-86.0 | 33-80 | 23.5-92.1 | 47-79 | 58-73 |
| Performance status 0-1 (%) | Unknown | 96 | Unknown | 92.4 | 92.8 | 84 | 58.7 | 90.2 | 91.8 | 90.6 | 90.2 |
| Postmenopausal (%) | Unknown | Unknown | Unknown | 82.2 | Unknown | 79.7 | 80.4 | 83.9 | 81.6 | 73.1 | Unknown |
| PR positive (%) | Unknown | Unknown | 82.4 | Unknown | Unknown | Unknown | Unknown | 79.5 | 81.4 | 81.3 | Unknown |
| De novo metastasis (%) | 23.2 | 40 | 23.3 | Unknown | 25 | 20.7 | 94.3 | 24.3 | 26.8 | 24.3 | 46 |
| Visceral metastases (%) | Unknown | 47 | 36.7 | 81.4 | 72.2 | 29 | 51.6 | 47.5 | 51 | 53.8 | 57.3 |
| Sensitivity to endocrine therapy (%) | Unknown | Unknown | Unknown | Unknown | Unknown | Unknown | Unknown | Unknown | 70.3 | 52.2 | Unknown |
| Prior endocrine lines (median) | 4 | Unknown | Unknown | 4 | 3 | 0 | 0 | 3 | 0 | 1 | 0 |
| Total N | 82 | 70 | 411 | 118 | 126 | 128 | 281 | 219 | 310 | 182 | 772 |
| Fulvestrant N | 3 | 0 | 158 | 56 | 0 | 46 | 48 | 87 | 103 | 92 | 0 |
| AI N | 75 | 70 | 253 | 57 | 0 | 82 | 233 | 110 | 207 | 90 | 772 |
| Letrozole N | 73 | 0 | 226 | / | 126 | 79 | / | / | 195 | 90 | 772 |
| Anastrozole N | 1 | 0 | / | / | 0 | / | / | / | 9 | 0 | 0 |
| Exemestane N | 1 | 0 | / | / | 0 | / | / | / | 3 | 0 | 0 |
| Tamoxifen N | 2 | 0 | 0 | 5 | 0 | 0 | 0 | 8 | 0 | 0 | 0 |
| 1^st^ line N | 0 | 70 | 147 | 0 | 14 | 76 | 236 | 0 | 225 | 61 | 772 |
| 2^nd^ line N | 0 | 0 | 107 | 0 | 18 | 19 | 45 | 0 | 85 | 51 | 0 |
| ≥3^rd^ line N | 82 | 0 | 157 | 118 | 94 | 33 | 0 | 219 | 0 | 70 | 0 |

**References**

1. Hoste G, Punie K, Wildiers H, Beuselinck B, Lefever I, Van Nieuwenhuysen E, et al. Palbociclib in highly pretreated metastatic ER-positive HER2-negative breast cancer. Breast cancer research and treatment. 2018;171(1):131-41.

2. Wilkie J, Schickli MA, Berger MJ, Lustberg M, Reinbolt R, Noonan A, et al. Progression-Free Survival for Real-World Use of Palbociclib in Hormone Receptor-Positive Metastatic Breast Cancer. Clinical breast cancer. 2020;20(1):33-40.

3. Varella L, Eziokwu AS, Jia X, Kruse M, Moore HCF, Budd GT, et al. Real-world clinical outcomes and toxicity in metastatic breast cancer patients treated with palbociclib and endocrine therapy. Breast cancer research and treatment. 2019;176(2):429-34.

4. Battisti NML, Kingston B, King J, Denton A, Waters S, Sita-Lumsden A, et al. Palbociclib and endocrine therapy in heavily pretreated hormone receptor-positive HER2-negative advanced breast cancer: the UK Compassionate Access Programme experience. Breast cancer research and treatment. 2019;174(3):731-40.

5. Brufsky A, Mitra D, Davis KL, Nagar SP, McRoy L, Cotter MJ, et al. Treatment Patterns and Outcomes Associated With Palbociclib Plus Letrozole for Postmenopausal Women With HR(+)/HER2(-) Advanced Breast Cancer Enrolled in an Expanded Access Program. Clinical breast cancer. 2019;19(5):317-25.e4.

6. Petracci F, Abuin GG, Pini A, Chacón M. RENATA study-Latin American prospective experience: clinical outcome of patients treated with palbociclib in hormone receptor-positive metastatic breast cancer-real-world use. Ecancermedicalscience. 2020;14:1058.

7. Lin J, McRoy L, Fisher MD, Hu N, Davis C, Mitra D, et al. Treatment patterns and clinical outcomes of palbociclib-based therapy received in US community oncology practices. Future oncology (London, England). 2021;17(9):1001-11.

8. Manso L, Hernando C, Galán M, Oliveira M, Cabrera MA, Bratos R, et al. Palbociclib combined with endocrine therapy in heavily pretreated HR(+)/HER2(-) advanced breast cancer patients: Results from the compassionate use program in Spain (PALBOCOMP). Breast. 2020;54:286-92.

9. Porte B, Carton M, Lerebours F, Brain E, Loirat D, Haroun L, et al. Real life efficacy of palbociclib and endocrine therapy in HR positive, HER2 negative advanced breast cancer. Breast. 2020;54:303-10.

10. Palumbo R, Torrisi R, Sottotetti F, Presti D, Rita Gambaro A, Collovà E, et al. Patterns of treatment and outcome of palbociclib plus endocrine therapy in hormone receptor-positive/HER2 receptor-negative metastatic breast cancer: a real-world multicentre Italian study. Therapeutic advances in medical oncology. 2021;13:1758835920987651.

11. DeMichele A, Cristofanilli M, Brufsky A, Liu X, Mardekian J, McRoy L, et al. Comparative effectiveness of first-line palbociclib plus letrozole versus letrozole alone for HR+/HER2- metastatic breast cancer in US real-world clinical practice. Breast cancer research : BCR. 2021;23(1):37.
